# Dhaka city water logging hazards: area identification and vulnerability assessment through GIS-remote sensing techniques

**DOI:** 10.1007/s10661-023-11106-y

**Published:** 2023-04-05

**Authors:** Rafiul Alam, Zahidul Quayyum, Simon Moulds, Marzuka Ahmad Radia, Hasna Hena Sara, Md Tanvir Hasan, Adrian Butler

**Affiliations:** 1 BRAC James P Grant School of Public Health, BRAC University, 6th Floor, Medona Tower, 28 Mohakhali Commercial Area, Bir Uttom A K Khandakar Road, Dhaka, 1213 Bangladesh; 2 Imperial College, South Kensington Campus, London, SW7 2AZ UK

**Keywords:** Water logging, GIS-RS, Spatio-temporal, Dhaka city

## Abstract

Water logging is one of the most detrimental phenomena continuing to burden Dhaka dwellers. This study aims to spatio-temporarily identify the water logging hazard zones within Dhaka Metropolitan area and assess the extent of their water logging susceptibility based on informal settlements, built-up areas, and demographical characteristics. The study utilizes integrated geographic information system (GIS)-remote sensing (RS) methods, using the Normalized Difference Vegetation Water and Moisture Index, distance buffer zone from drainage streams, and built-up distributions to identify waterlogged zones with a temporal extent, incorporating social and infrastructural attributes to evaluate water logging effects. These indicators were integrated into an overlay GIS method to measure the vulnerability level across Dhaka city areas. The findings reveal that south and south-western parts of Dhaka were more susceptible to water logging hazards. Almost 35% of Dhaka belongs to the high/very highly vulnerable zone. Greater number of slum households were found within high to very high water logging vulnerable zones and approximately 70% of them are poorly structured. The built-up areas were observed to be increased toward the northern part of Dhaka and were exposed to severe water logging issues. The overall findings reveal the spatio-temporal distribution of the water logging vulnerabilities across the city as well as its impact on the social indicators. An integrated approach is necessary for future development plans to mitigate the risk of water logging.

## Introduction

Bangladesh is undergoing environmental degradation due to rapid urbanization, increasing population growth, and rapid industrialization (Tawhid, 2004a, b). Rapid urbanization is linked with economic development, which has an increasingly higher contribution to the national economy, with almost 36% of the gross domestic product (GDP) coming from the urban economy (Hussain, 2013). However, when the growth of the urban population takes place at an exceptionally rapid rate, people and governments face difficulties in keeping pace with the changing situations due to resource constraints and the inability to manage and respond quickly (Bari & Hasan, 2001). The country has also been exposed to several types of climate-induced hazards including variations in temperature, excessive and erratic rainfall, water logging, and flooding which adversely affect urban life and livelihoods (Rabbani et al., 2011). The situation gets worse when climatic phenomena become tied with non-climatic factors including population density, poverty, rural–urban migration, illiteracy, unplanned urbanization, poor management of natural resources, and lack of public utilities and services (Adri, 2013).

Dhaka, the capital of Bangladesh, has been ranked top in terms of urban population density (Demographia World Urban Areas, 2020). Due to the speedy industrialization and urbanization process, the city is among the top 5 fastest growing megacities in the world (United Nations, 2018) and at the same time a development hub for Bangladesh. However, this development is also bringing in several adverse impacts such as deterioration of environmental quality, increased air and water pollution, and congestion. Dhaka city is also experiencing several socio-economic problems such as rising inequality, poverty, inadequate social security, and corruption among others. Water logging, traffic congestion, improper solid waste disposal, black smoke emission from vehicles and industry, air and noise pollution, and water pollution from industrial discharge are also very common problems of the city (Tawhid, 2004a, b). In recent times, water logging has become one of the main causes for apprehension damaging infrastructures, disrupting daily lives, and demolishing vegetation and aquatic habitats. Several initiatives undertaken by WASA (Water and Sewerage Authority) and the two City Corporations to improve the existing condition have failed due to the absence of proper urban design and planning, landscape architecture, and most importantly lack of coordination between project activities and stakeholders (Subrina & Chowdhury, 2018).

Urban water logging disaster refers to the phenomenon when a rainstorm or short-time heavy rain surpasses the capacity of the urban drainage system (Xue et al., 2016), a very common situation during rainy season in Dhaka. Water logging in Dhaka has become an increasingly predominant burden for the city dwellers and is creating adverse social, physical, economic, and environmental consequences by disrupting regular life, causing traffic paralysis and infrastructural damage, and destruction of flora and fauna (Subrina & Chowdhury, 2018). Urban infrastructures including low-lying houses, schools, colleges, shops, and business premises are greatly affected by the water logging problem. People of low-income groups, particularly grocery shop owners, vegetable vendors, and day laborers, are the main sufferers (Majumder et al., 2018). In addition to the water logging problem, previous studies have shown that almost every year about 60% of Dhaka city becomes submerged due to flooding by the Balu River in the east and the Tongi Khal in the north (Gain et al., 2015). While actions have been taken to ease the growing problem of fluvial flooding and water logging (Papry & Ahmed, 2015), these have largely been inadequate. Substantial increase in the impervious area and improper solid waste management obstruct the natural drainage pattern leading to a shortening of the runoff concentration time and an increase of the peak flow (Mowla & Islam, 2013). Most drains in Dhaka city are clogged by solid waste and plastic waste, due to irregular cleanup, improper management, and poor littering and fly-tipping by city dwellers (Anik, 2019). Excessive rainfall, disappearance of the natural drainage system, and lower capacity of the drainage system are considered the main reasons behind this.

The highest rainfall in Dhaka was in 2017 which was 2892 mm and the lowest was 1329 mm in the year 2012 (Chakraborty, 2019). Besides, the average rainfall in winter is around 35.3 mm compared to 1353.6 mm in monsoon and the annual average rainfall is 2059.9 mm (Mahbub et al., 2021). Furthermore, unplanned city development, uncontrolled silt load arising from the construction works, and major road works involving huge digging during the rainy season further worsen the situation (Tawhid, 2004a, b). According to the Bangladesh Institute of Planners (BIP), from 2011 to 2019, minimum 3483 acres of water bodies and lowlands across the metropolitan area in Dhaka have been loaded up (Nabi, 2019). However, the city lacks adequate retention and detention capacity of rainwater and sustainable development of the drainage system during urban planning and design (Mowla & Islam, 2013). Several studies have addressed water logging hazards and risks of south-west and south-east coastal zones, at different river basins of Bangladesh adapting mix-method approach and GIS-remote sensing techniques (Hassan & Mahmud-ul-islam, 2014; Rahman et al., 2009; Tareq et al., 2018; Islam et al., 2020; Huda et al., 2022). However, to the best of our knowledge, no studies have evaluated water logging hazards through the lens of GIS and remote sensing integrated approach and have not emphasized the contribution of rapid urbanization. This study has incorporated several techniques to identify water logging hazards using historical data with GIS and remote sensing techniques coupled with rapid urbanization to identify the water logging hazard zone.

This study attempts to delineate the surface waterlogged area by analyzing spatio-temporal data of 1990, 2010, and 2019 through establishing a GIS-remote sensing–based spatial model. After identifying the waterlogged hazard areas, the study also aims to assess the vulnerability in those identified water logging hazard zones based on the spatial distribution of built-up areas, slums,^1^ dwelling housing types, and population density and rank them based on predetermined scales.

## Materials and methods

**Fig. 1:**
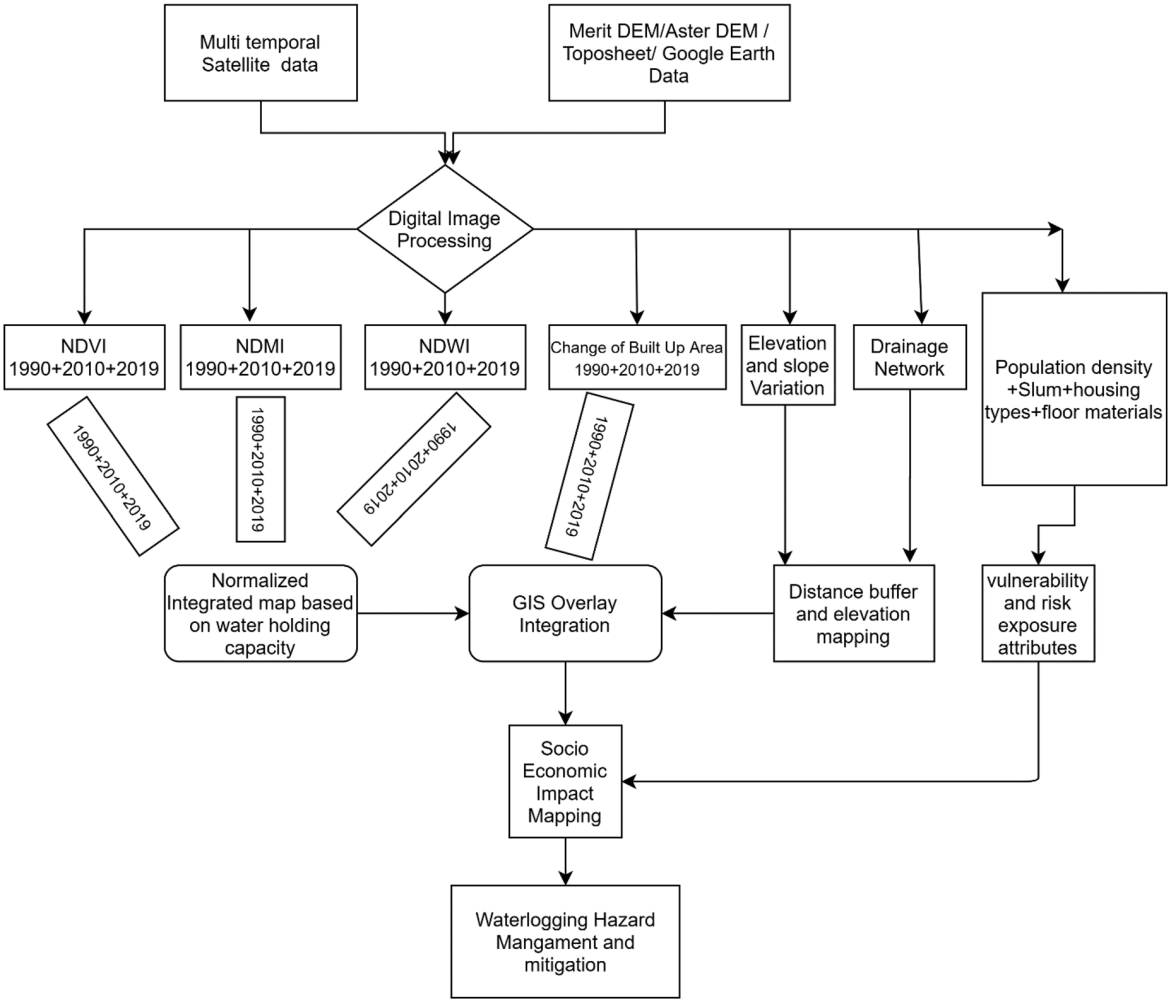
Flow diagram of research and data integration method

This study was conducted in two steps. First, we utilized different spatial hazard attributes using multi-temporal Landsat and Sentinel satellite imagery. We combined images from 1990, 2010, and 2019 with topographical data including slope, elevation, and drainage network to detect the water logging hazard zones. Then, we merged the social attributes with spatial data of the hazard zones across Dhaka city to assess the exposure and vulnerability of Dhaka residents to water logging. Data collection and analysis workflow are elaborated in Fig. 1.

### Identifying the surface water logging hazard areas

We developed an overlay model to identify the surface water logging hazard zones. An overlay model is a GIS operation that superimposes multiple data points to identify relationships between them. For deploying this model, Normalized Difference Vegetation Index (NDVI), Normalized Difference Water Index (NDWI), and Normalized Difference Moisture Index (NDMI) were analyzed for the years 1990, 2010, and 2019 for Dhaka city. Topographical data such as slope and elevation, and buffer drainage distance were determined by using MERIT Hydro digital elevation data which is a new global flow direction map at 3 arc-second resolution (~ 90 m at the equator) derived from the latest elevation data (MERIT DEM) and water body datasets (G1WBM, GSWO, and OpenStreetMap). The corresponding variables (NDVI, NDWI, NDMI, topographical variation and drainage density, and built-up areas) were initially assigned with equal weight. Then, an overlay was generated by adjusting the rank and weight for mapping the water logging hazard zones.

#### Normalized Difference Vegetation Index

Normalized Difference Vegetation Index (NDVI)^2^ is a globally accepted remote sensing index widely used to detect the vegetation, forest extension, and the water bodies over the surface using red and near-infrared light (Jackson & Huete, 1991; Sahu, 2014; Tucker, 1979). An NDVI value always ranges from −1 to +1. A value of +1 indicates dense vegetation, while −1 implies the presence of extensive deep-water bodies, with 0 signifying the absence of any vegetation. For this study, we followed the methods for segregating vegetated areas from water-logged areas used by Dwivedi and Sreeniwas (2002) who measured NDVI value of 0.13 as a threshold using Landsat MSS and TM, and IRS-1A LISS-I data.

#### Normalized Difference Water Index

The Normalized Difference Water Index (NDWI) examines water content pixels analogous to the way NDVI measures vegetation (Geogr et al., 2003; Gao, 1996). The values of the NDWI range from −1 to +1 (McFeeters, 1996). According to Chowdary et al. (2008), NDWI for waterlogged areas ranges from 0 to +1 where +1 indicates the existence of deep waterbodies with 0 for non-waterbodies. Multi-temporal satellite imageries were used to generate the NDWI by using the formula^3^ used in the study by McFeeters (1996).

#### Normalized Difference Moisture Index

NDMI^4^ is a modified version of NDWI which is theoretically a similar measure of the previous indices that refers to the spatial variation of surface moisture and wetness (Wilson & Sader, 2002). Higher values of NDMI indicate high soil moisture and low values denote low soil moisture content. According to Wilson and Sader (2002) and Goodwin et al. (2008), an NDMI of more than +0.20 indicates moist soil surface with the very good potentiality of groundwater and from +0.20 to +0.10 indicates wet to dry soil with moderate potentiality.

These three indices were analyzed in the GIS environment using raster calculator tools^5^ (Sar et al., 2015). After downloading the satellite images, all the necessary corrections, band composition, and masking were accurately completed prior to analysis. Radiometric correction was applied including haze and noise reduction with histogram equalization using Erdas Imagine software. Reprojection tool was used to project all images into World Geodetic System (WGS) 1984. After that, all the essential seven bands of satellite images were composed into one single image using data management tool. Subsequently, the cells were extracted from the composed image corresponding to the study area using extract by mask tool in GIS environment.

#### Topographical variation in terms of elevation and slope

Topographical variation was considered a significant indicator for water logging analysis. Elevation and slope data of 2019 were derived from the MERIT DEM images as topographical variables. Higher altitudes are associated with a lower probability of water logging, while lower altitudes are more susceptible to water logging. A lower topographic slope allows the landscape to retain water and cause water logging, while a steep slope drains quicker.

#### Drainage buffer distance

The drainage buffer distance was used as a proxy to establish the spatial relation between the drainage network and waterlogged areas. Shapefiles of lake and large water bodies were collected from the Survey of Bangladesh dataset to determine the area boundary and their extent. Survey of Bangladesh has been providing spatial data of natural features, services and utilities, transport network, distribution of industries, etc. After that, a distance tool in spatial analysis was applied. Sahu (2014) conducted a study on mapping of waterlogged areas in Moyna Basin of West Bengal. He found that high density of waterlogged areas is positively correlated with high canal density. Another study found a higher water logging probability for areas close to the canals (Sar et al., 2015). Therefore, the drainage density and their proximity to a particular buffer zone can be considered potential determinants of waterlogged vulnerable areas.

#### Changes in built-up areas

The built-up area was measured using Landsat and Sentinel multispectral satellite imageries through unsupervised classification. The unsupervised classification used a grouping algorithm which can automatically determine the frequently repetitive texture patterns to detect built-up areas (Gowthami & Thilagavathi, 2014). The analysis was simplified with two likelihood classes: built-up area pixels and other pixels. The temporal variation of the built-up area shows that its density has been increasing continuously since 1990. In this study, built-up area includes all infrastructure, residential, commercial, mixed-use and industrial areas, villages, settlements, road networks, pavements, and man-made structures. These infrastructures make the surface impervious, both increasing the amount of surface runoff and collecting water in surface depressions. The situation is exacerbated by the lack of effective drainage system in the city.

All the above indicators are considered input variables to the overlay model. After assigning these variables to equal weights and rank, the overlay analysis was applied to identify the water logging hazard zone.

### Water logging vulnerability assessment with social attributes

For generating vulnerability index, slum distribution, population density, dwelling house types, and its floor materials along with changing trend of built-up areas from 1990 to 2019 has been considered. The slum distribution map was generated from slum census 2014 data and BRAC urban slum web portal. The population density map was developed from the global human settlement dataset. Housing characteristics and floor materials data were derived from the Slum census survey 2014 dataset. Built-up areas were extracted from the Landsat and Sentinel satellite imageries. Dot density method was applied to represent the spatial distribution of social attributes over the water logging hazard zones. Categorical values of the social attributes were represented by dots over the identified waterlogged areas to quantify their extent. Each dot represents a particular number of group values and the density of the dots reflected the status of that attribute.

Finally, an integrated spatial model was used to map the water logging hazard and socio-economic risk level using GIS environment over the entire Dhaka city with the aim to provide useful information for better urban planning, management and mitigation of the urban water logging hazard along with its risk factors. For instance, identification of poorly structured households within water logging hazard zones may be prioritized while planning development initiatives. The sources of data that have been used are given in Table 1.

**Table 1:** Data sources of the variables used

**Data**	**Source**	**Derived information**
Digital elevation model	MERIT Hydro: global hydrography datasets, 2019	Elevation, slope and contour data, distance from drainage
Multi-temporal satellite images	NASA Earth data and USGS, Sentinel and Landsat temporal satellite imageries (1990, 2010, 2019)	NDWI, NDVI, NDMI
Existing surface water bodies	Survey of Bangladesh, 2018, 2019	The distance of areas from the existing lake and large water bodies
Population density	Global human settlement, 2018	Population density of different areas in Dhaka city
Slum distribution	BRAC urban slum amp and Slum Census 2014	Slum households’ distribution map
Housing types and floor materials	Slum census 2014	Floor materials for dampness and housing types

## Results

### Water logging hazard attributes

#### Normalized Difference Vegetation Index

**Fig. 2:**
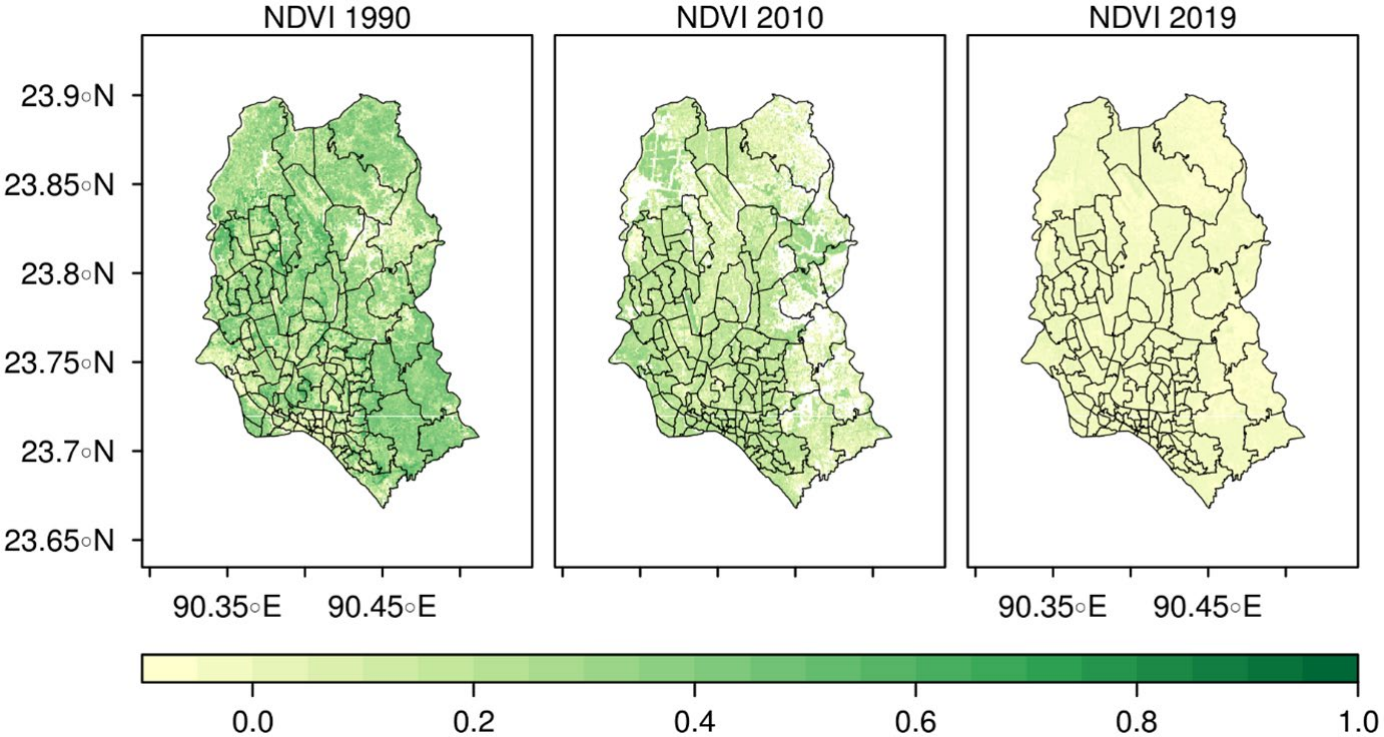
Temporal distribution of the NDVI map of Dhaka city in 1990, 2010, and 2019 respectively

In the present study, NDVI technique on multi-temporal data has been used for the identification of water-containing pixels. Figure 2 depicts the NDVI map for 1990, 2010, and 2019. In 1990, the ranges of NDVI were found to be greater than 0.2 which indicates moderate to high vegetation coverage in some areas. On the other hand, in 2010, the NDVI value became lower than 0.2 indicating lower vegetation coverage than 1990. In 1990, the city had a large number of vegetation coverage which were later covered up by land due to the higher growth rate of population and rapid urbanization. Later in 2019, the vegetation coverage declined sharply and made the city more vulnerable to water logging. The value of NDVI reveals that the city did not have much dense forest cover since 1990, while the NDVI value in 2010 and 1990 represents the agricultural land. In 1990, the city had a large coverage of natural and artificial canals vegetation compared to other years. Subsequently, in 2010, rapid urbanization took place, which slightly decreased greeneries. This notion also supports the study findings of Rahman et al. (2011), where they found Dhaka obtained copious vegetation from the 1989 to 2010 period. However, from the measured values of both vegetation coverage, greeneries and water bodies have declined drastically in 2019, as shown in Fig. 2

#### Normalized Difference Water Index

**Fig. 3:**
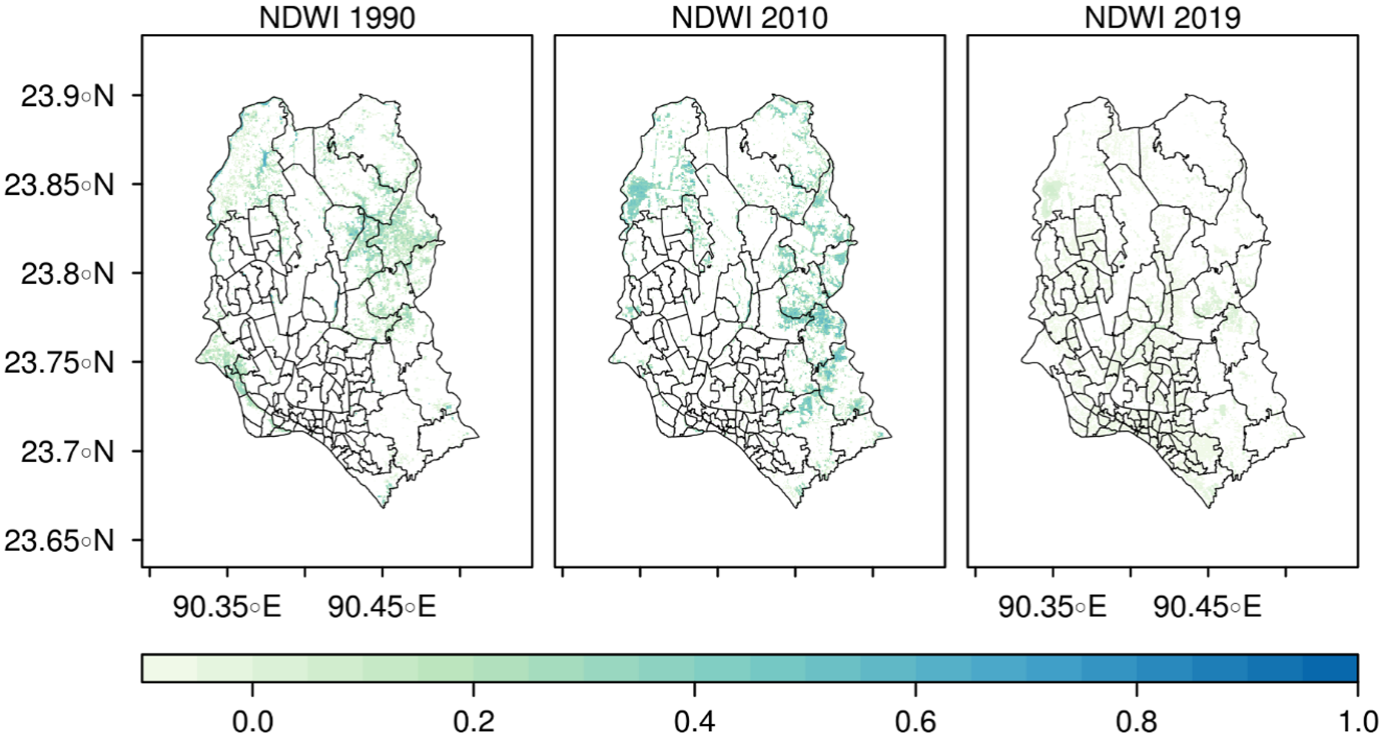
Temporal distribution of the NDWI map of Dhaka city in 1990, 2010, and 2019

The NDWI map illustrated in Fig. 3 shows the temporal variation of NDWI value of the years of 1990, 2010, and 2019. The highest value of NDWI was observed in 1990 and 2010. In 2019, the highest value of NDWI was observed as 0.12 since urbanization and expansion of built-up areas took place, which was also responsible for lowering water content in 2019. According to Chatterjee et al. (2005), the NDWI value ranging from −0.34 to +0.59 represents some water-logged areas. Nevertheless, in Dhaka city, poor drainage networks and other anthropogenic factors have influenced the value of the index.

#### Normalized Difference Moisture Index

**Fig. 4:**
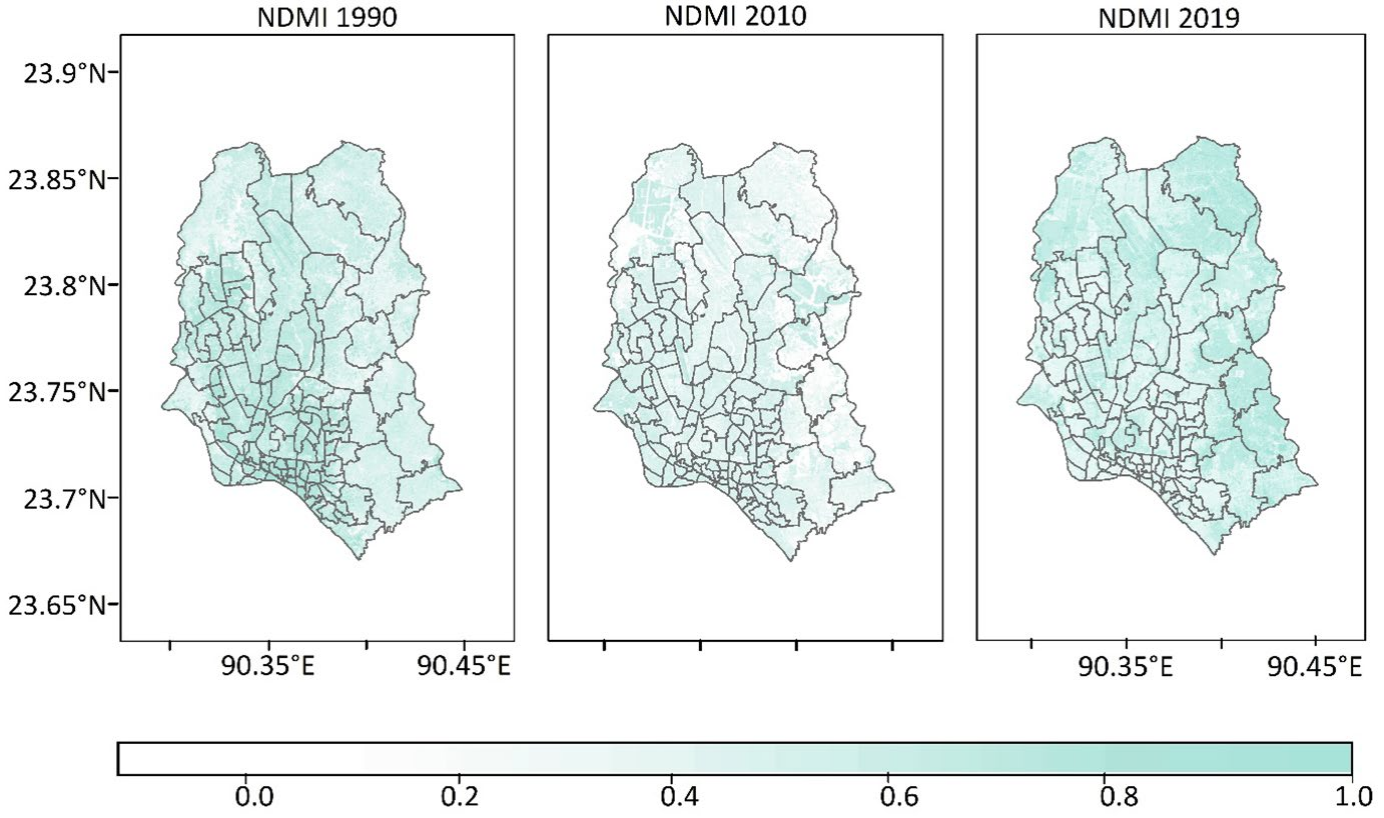
Temporal distribution of the NDMI map of Dhaka city in 1990, 2010, and 2019 respectively

In NDMI, the higher values of +0.85 indicate the existence of more soil moisture under massive water bodies and lower values of +0.1 indicate low soil moisture content, as depicted in Fig. 4. It shows the temporal variation of NDMI where the value ranges of NDMI, indicating that the soil in 1990 was highly moist, which has come from the surface runoff and might have resulted from the rainfall; in 2010, value ranges of NDMI manifest the soil was comparatively dry, whereas the highest ranges of value around +0.39 indicate the soil had higher level of moisture in 2019 than in 2010. However, the observed soil moisture in the middle part of Dhaka city reflected susceptibility to water logging.

According to FAO (1988), Dhaka is located in Deep Red Brown Terrace soil region. This type of soil is moderately well drained, reddish to yellow–brown, and strongly to extremely acidic. Besides, the soil texture is silty loam which is predominant on the Meghna estuarine floodplain. As for silty soil, it tends to have moist soil contents. The NDMI values also found the soil moisture in Dhaka city, indicating the likelihood of natural water logging prevailing is high in Dhaka city.

#### Topographic and slope variation in term of DEM model

**Fig. 5:**
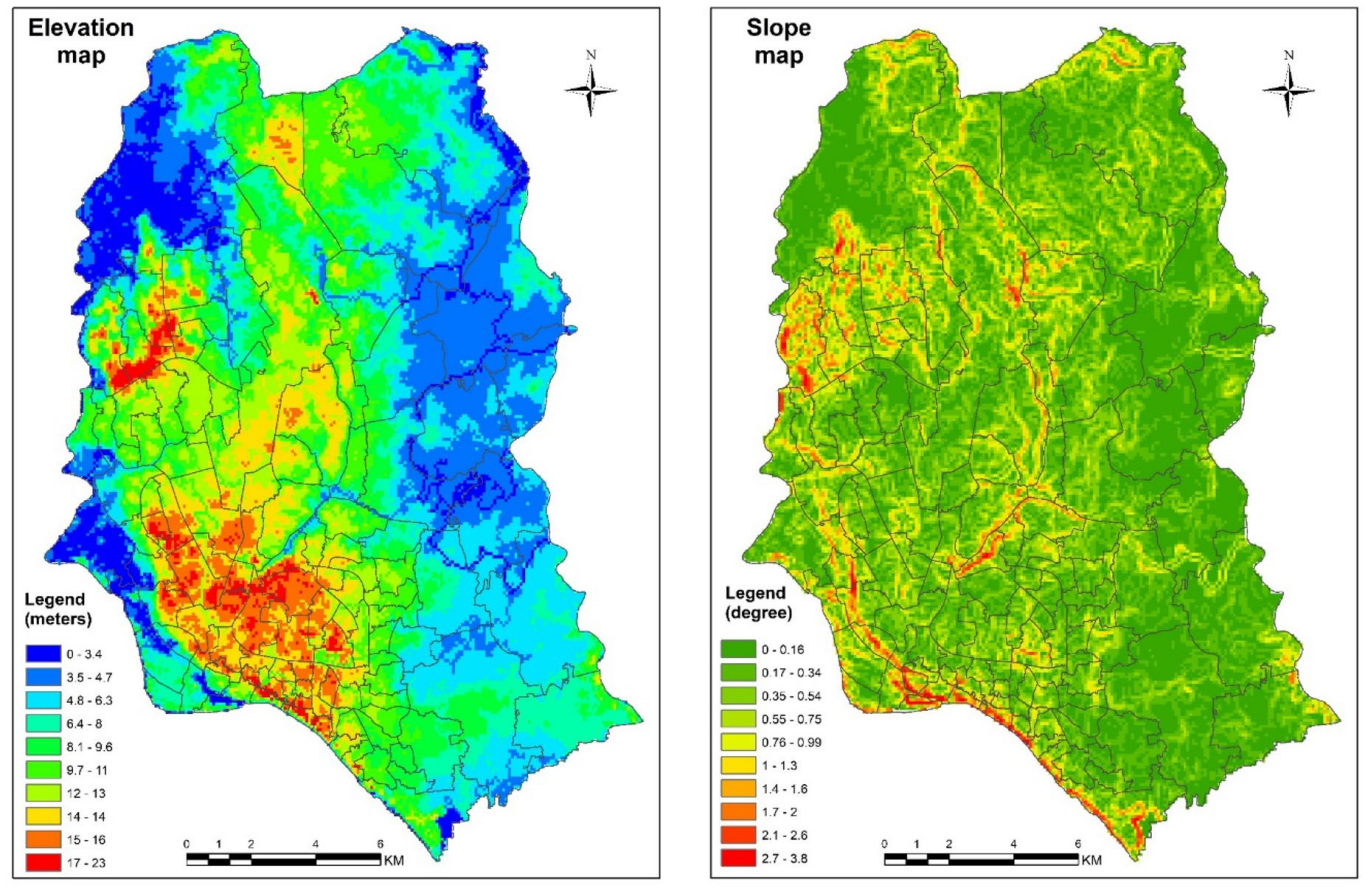
Elevation and slope distribution map of Dhaka city

Topographic variation is an important factor for the waterlogged situation in flat terrain (Kaiser et al., 2013). The elevation map and slope map of the study region have been generated from the Multi-Error-Removed Improved-Terrain (MERIT) DEM satellite data. The highest altitude observed from the analysis is 23 m. The lowest altitude of 0–10 m indicates highest waterlogged zones in the study site which lies in the northeastern part of Dhaka city. Besides, the slope of the study area varies from 0.16 to 3.8° (Fig. 5). Areas with high slope and higher altitudes are affected comparatively less by water logging while areas of lower altitude are highly susceptible to the water logging phenomenon.

#### Distance buffer zone from the drainage stream

**Fig. 6:**
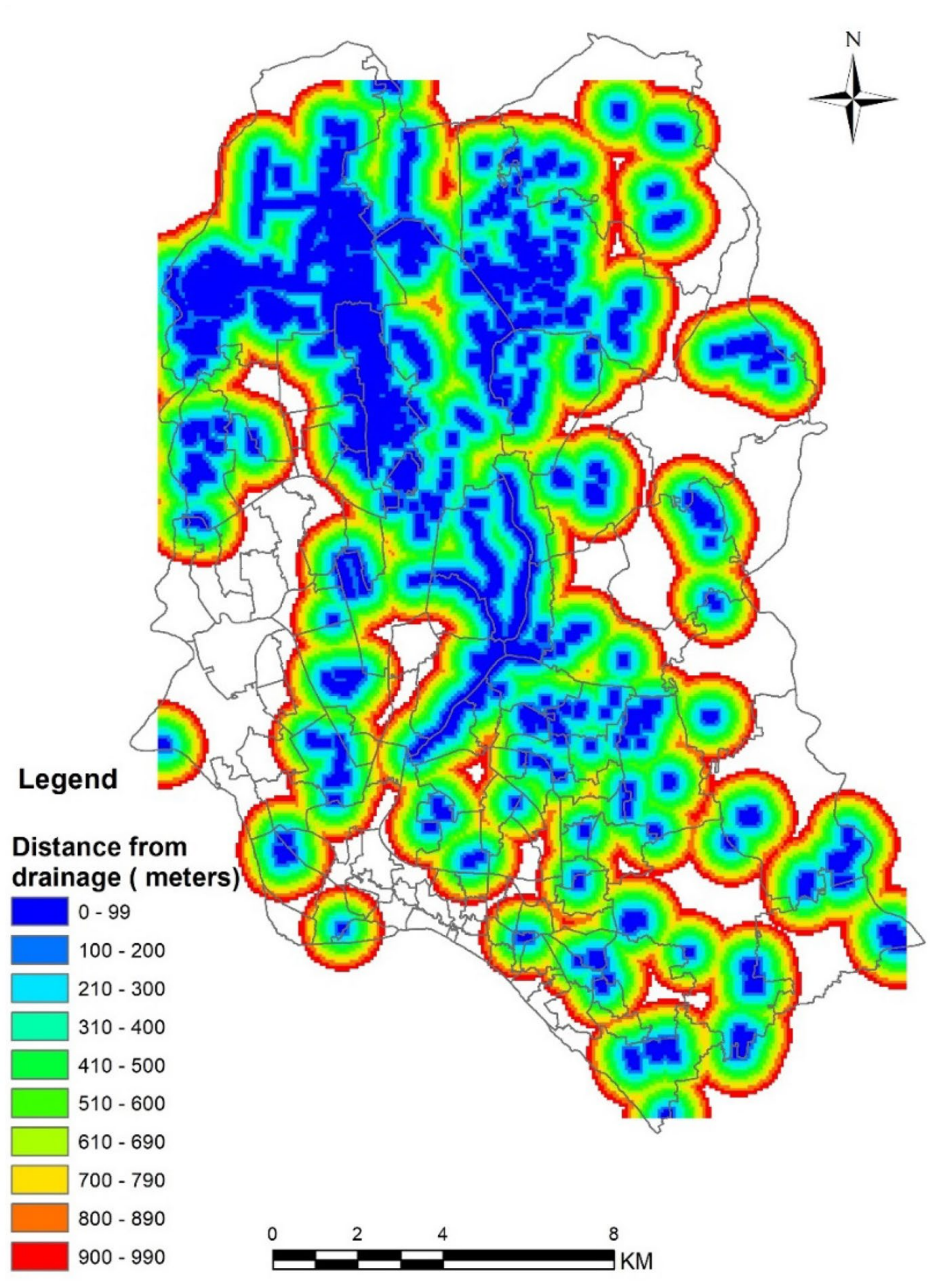
Distance buffer from the streams in Dhaka city

In this study, the drainage buffer distance was used to establish the spatial relationship with the existing natural drainage municipality network. In Fig. 6, the distance from drainage ranges from 0 to 990 m. Closer proximity of area from a particular stream indicates a lower possibility of water logging as the water runs off to the nearest stream, e.g., lakes and canal, quickly.

In particular, during monsoon season, improperly treated drainage is a major cause of water logging. Besides, half of the drains in the city are not covered, and it is common to find them clogged with garbage (Kabir et al., 2018). Dhaka has 12 drainage catchment zones depending on the drainage network (Tawhid, 2004a, b). WASA provides 38% of the city’s drainage and sewerage network. Open canals, box culverts, storm sewer lines, and surface drain facilities make up the Storm Sewer Network. Aside from that, the natural drainage network drains around 80% of the water through canals and retention areas (Subrina & Chowdhury, 2018).

### Water logging vulnerable zone and influencing social attributes

#### Water logging and distribution of built-up areas

**Fig. 7:**
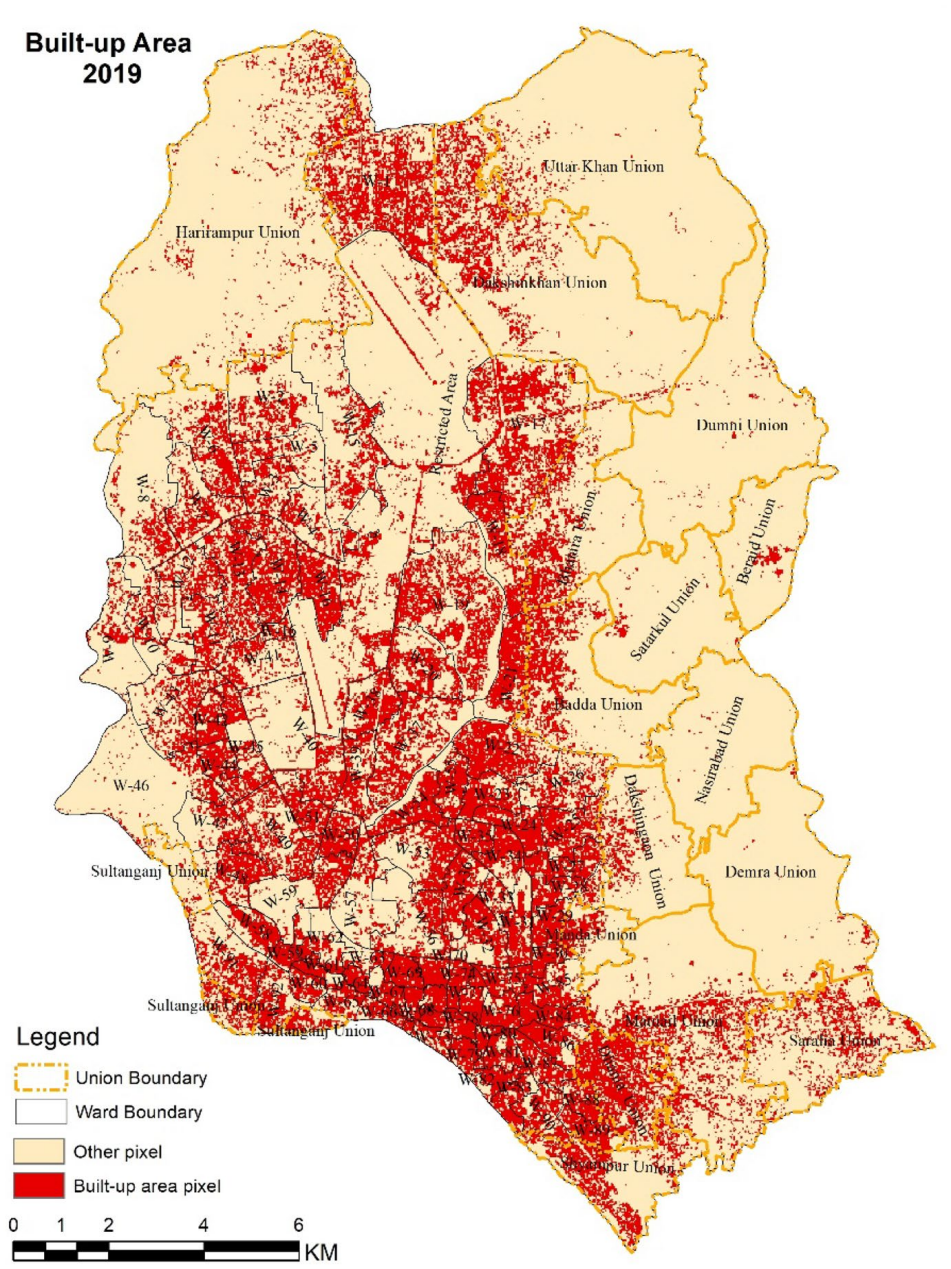
Built-up areas in Dhaka city in 2019

Built-up areas have been increasing due to rapid urbanization of Dhaka city. In 1990, the south part of Dhaka city, currently known as Dhaka South City Corporation, became more urbanized than other parts of Dhaka. Later in 2010 and 2019, the built-up areas spread over the middle and northern part of the Dhaka city. Figure 7 shows the distribution of built-up areas in the present day. Due to population growth and rural-to-urban migration, the trend of the population, as well as urbanization, has increased from 12% in 1990 to around 50% in 2011 (Grimm et al., 2008; United Nations, 2012). In 2019, larger areas of built-up zones were observed over most of Dhaka city. Therefore, the areas became highly vulnerable to becoming clogged down by water as the drainage network has not improved proportionately. On the contrary, the eastern part of the city, much of it brought within the jurisdiction of the greater Dhaka, is yet to develop and similar levels of built-up areas cannot be observed compared to the other areas.

#### Water logging and population density map

**Fig. 8:**
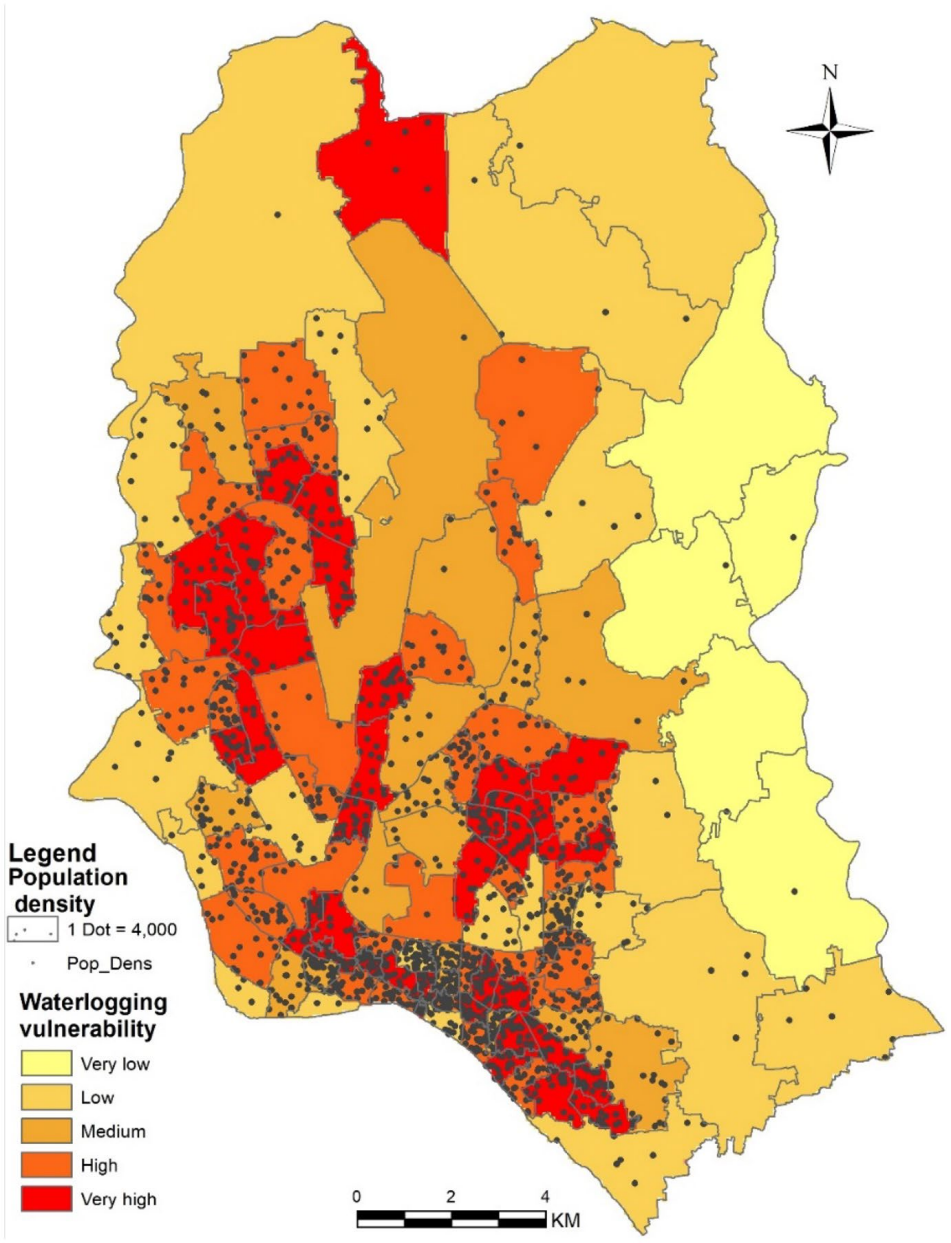
Water logging vulnerability zone and population density in Dhaka city

The extent of calamities associated with water logging will depend on the population density of the affected area. The population density map reflects the population densification in a particular area and its association with water logging. Figure 8 shows the water logging vulnerability zone and population density. In this figure, vulnerability zones are segmented in 5 different layers as very low, low, medium, high, and very high and these zones are associated with the existing population density. It was found that water logging vulnerability was very high in the wards having high population density. On the other hand, areas with medium to high water logging vulnerability also had high population density. In this map, medium vulnerable areas were found in Cantonment restricted area, Badda union, and some wards that had a decent population density. The map also shows that Hariramp union, Uttar khan union, Dakshinkhan union, Bhatara union, Dakshingaon union, Manda union, Matuail union, Sarail union, Shyampur union, Sultanganj union, and ward 8, 9, 33 were low water logging vulnerable areas and Dumni union, Beraid union, Satarkul union, Nasirabad union, and Demra union were very low water logging vulnerable areas with very small population density. In general, more areas with high population density are found to have a higher risk of water logging, the notable exception being the eastern and north-western part of the city which are mainly suburban areas surrounded by wetlands and rivers.

However, there has been rapid urbanization with increasing anthropogenic activities in Dhaka city, which has resulted in land cover changes over the years (Yao et al., 2015). Disparities in population distribution exist sharply in Dhaka. Some rich neighborhoods have a very small population density compared to neighborhoods with a high population density. The following Table 2 shows the Dhaka population and growth rate over the years from 1990. It depicts the population follows an increasing trend from 1990 to 2020.

**Table 2:** Dhaka population trend over the years

**Year**	**Population**	**Growth rate** (%)	**Growth**
2020	20,989,000	11.10	2,091,000
2017	18,898,000	7.40	1,300,000
2015	17,598,000	19.50	2,400,000
2010	14,731,000	19.50	2,400,000
2005	12,331,000	19.90	2,046,000
2000	10,285,000	23.40	1,953,000
1995	8,332,000	25.80	1,711,000
1990	6,621,000	42.10	1,961,000

Source: Bangladesh Bureau of Statistics—Dhaka Information Statistics; (Ullah & Islam, 2017)

#### Water logging and slum household distribution

**Fig. 9:**
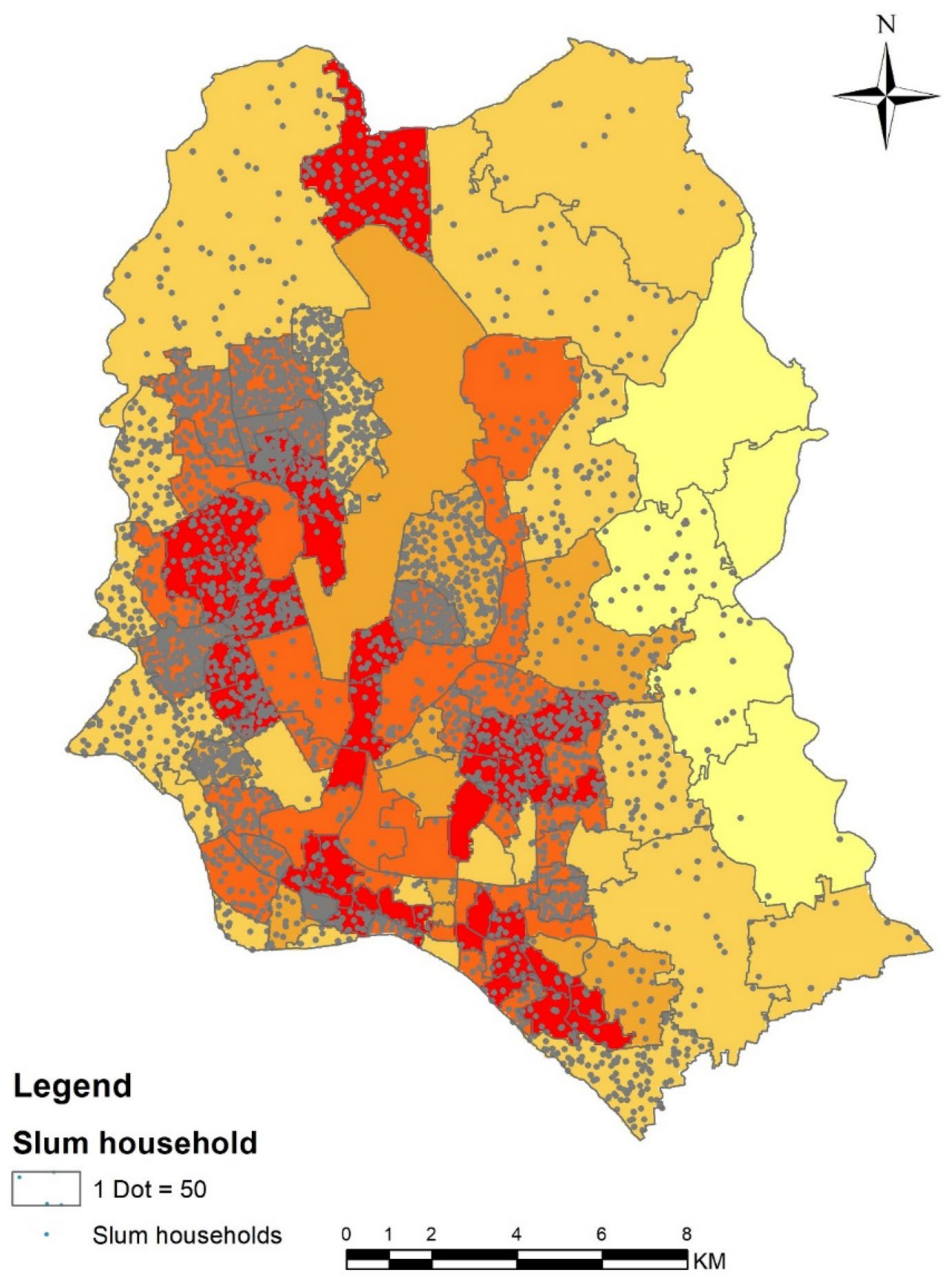
Water logging vulnerability zone and slum household’s distribution in Dhaka city

Slum population are one of the most vulnerable communities in the city living in informal settlements with poor living conditions. Their susceptibility level regarding water logging hazards are clearly depicted in the final generated map. Figure 9 depicts the dot density map of the slum households according to the Slum Census 2014 where 1 dot represents 50 households. The severity of water logging is high among the slum households because of the higher population density in slum areas, unplanned and unhygienic housing and sanitation systems, with very poor access roads and poor environment, etc. Figure 9 shows the water logging vulnerability zone and slum household distribution depict how many slum households are vulnerable to the problems of water logging. The vulnerability of water logging was very high among the wards with the existence of slum households. Also, the existence of slum households was common among the areas of high susceptibility of water logging such as wards (2, 5, 14, 7), which reveals that most of the slum households of Dhaka city are facing serious water logging problem.

#### Water logging and housing types of the dwellers

**Fig. 10:**
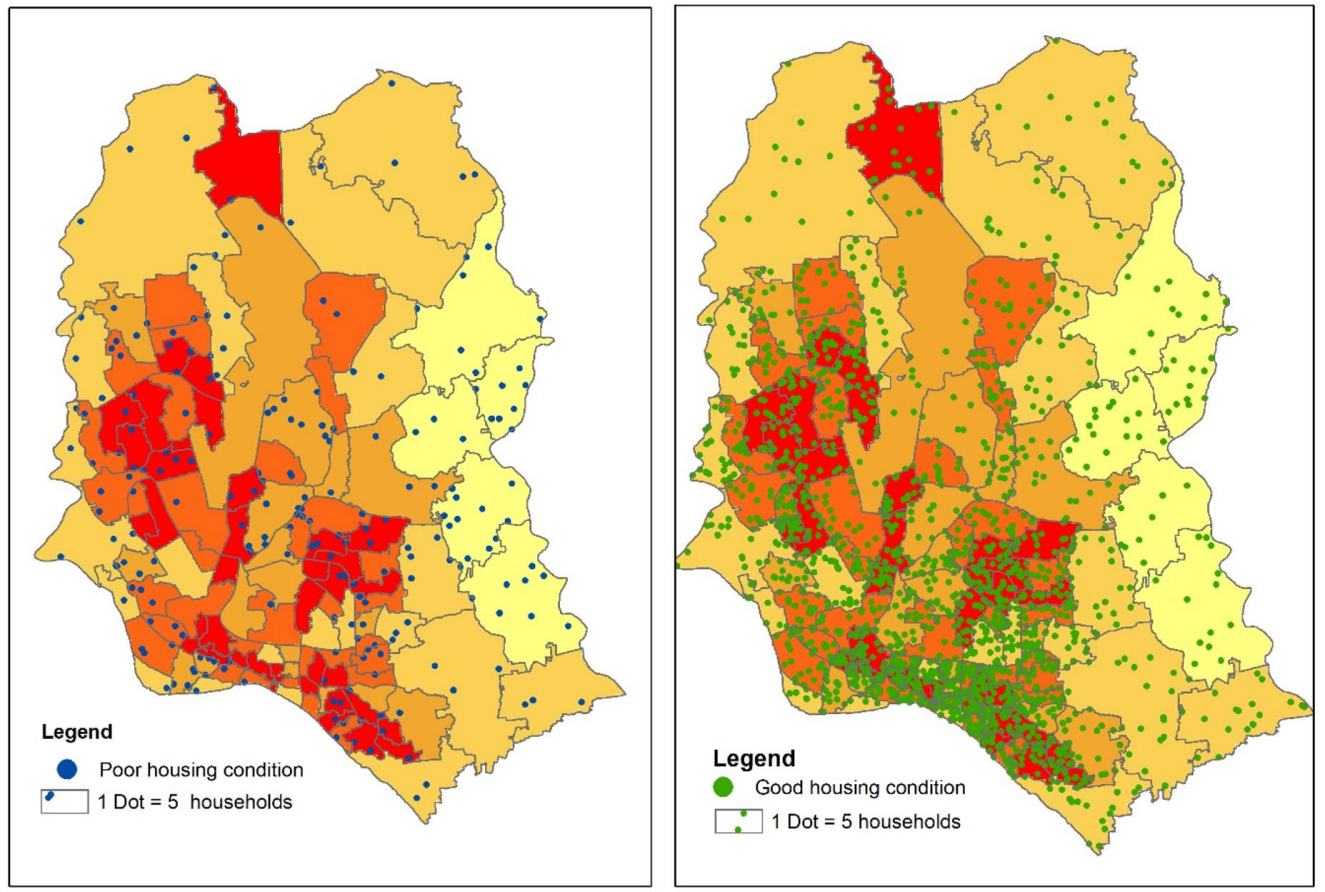
Water logging vulnerability zone and housing characteristics in Dhaka city

Among the housing types of the dwellers in Dhaka city, good housing (Pacca and Semi pacca)^6^ and poor housing condition (Jhupri and Kacca)^7^ have been categorized from the Slum Census 2014. The consequences and severity of the problem of water logging vary based on the housing types, making it necessary to consider housing types and associate that with water logging. Figure 10 depicts the housing types in the water logging vulnerable zones. It was found that water logging susceptibility was very high within the wards with good housing (Pacca and Semi Pacca). Within same wards, the vulnerability of water logging was also very high in the households with poor housing structures. The water logging susceptibility was very high among other wards with poor housing as well. Lastly, the following analysis implies that almost 70% of poorly structured households lies within high to very high water logging vulnerable zone.

#### Water logging and associated floor materials

**Fig. 11:**
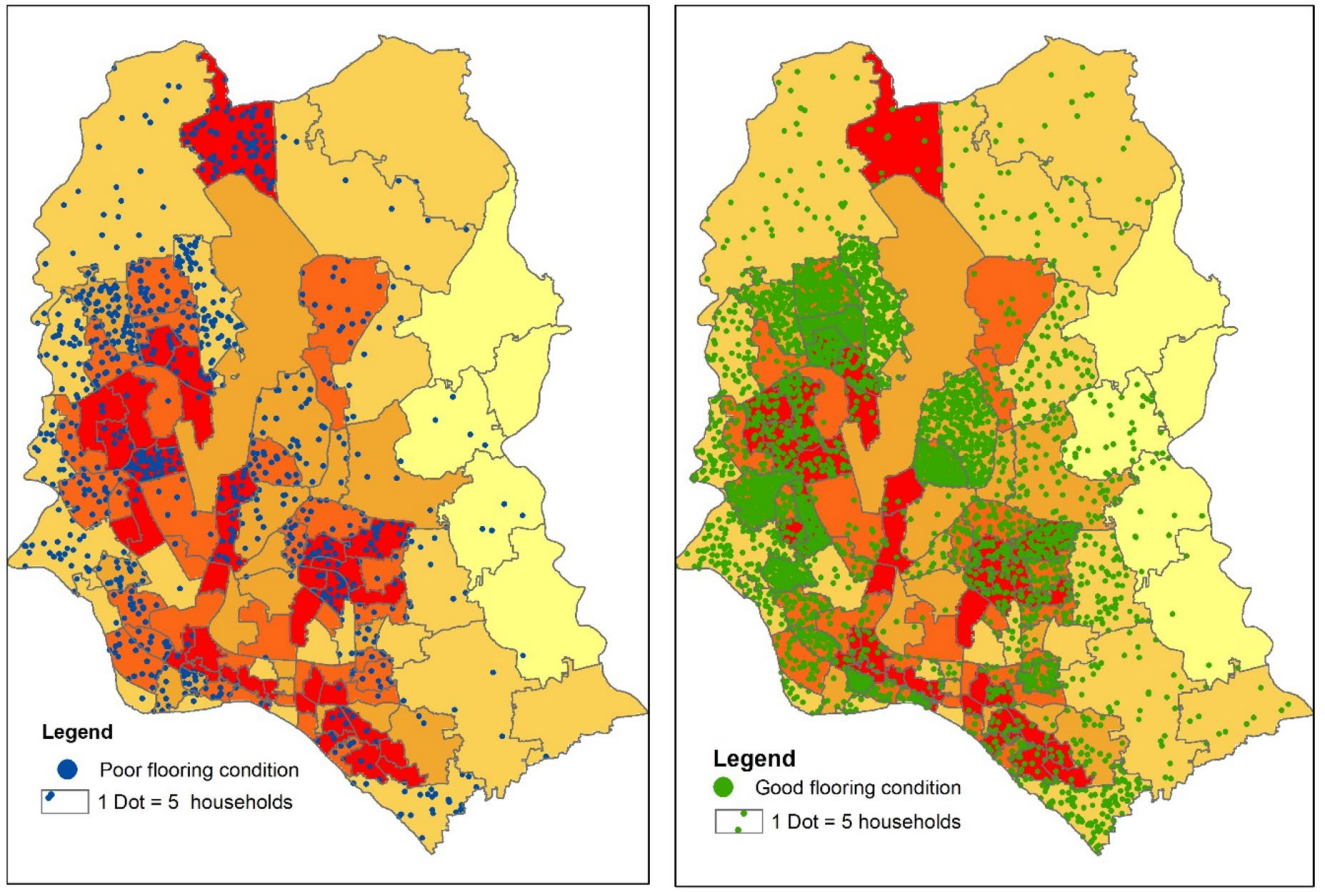
Water logging vulnerability zone and flooring condition in Dhaka city

A study on water logging conducted in the 4 urban areas (Sylhet, Dhaka, Mymensingh, and Chittagong) in Bangladesh found that 69% of houses in those areas became damaged due to water logging (Anisha & Hossain, 2014). That signifies that a poor housing structure in a water logging vulnerable zone will be more prone to experience adverse consequences, especially if floor materials of houses are made from poor construction materials. It can be assumed that water logging causes dampness in some floor materials, making it remain wet for a long period (Gazi & Hossain, 2019) and the issues of slipperiness or wetness occurred in the houses with mud floors. Figure 11 shows the water logging vulnerability zones and the poor and good flooring status in those areas. Bamboo and mud types floor were considered poor floor and the floor made of brick type materials belonged to good flooring condition. The existence of a huge number of houses with poor flooring was found in north and north-western part of Dhaka where the severity of water logging is also very high. A huge number of houses with good floor materials were found throughout Dhaka city, especially in ward 11, 12, and 13, where the vulnerability of water logging is also very high. As Dhaka is an urbanized city, most houses are made of brick; mud and bamboo-made floor was found in the peripheral areas of north, north-western part, and also in some slum areas in the middle part of Dhaka city where water logging vulnerability was also very high.

## Discussion

This study delineated the water logging vulnerable zone in a comprehensive way depicting the variables and indicators using an overlay weighted technique. The inclusion of temporal extension and social attributes made the research encompassing and exceptional. The method used the inclusion and integration of spatial, social, and demographic data for addressing water logging problem, make it unique, and will have significant contribution to advance scientific knowledge.

Moniruzzaman (2012), Tareq et al. (2018), and Awal (2014) conducted several studies on water logging in the south-west coastal region of Bangladesh. Severe flood, frequent cyclone events, tidal river mismanagement, and excessive rainfall were mentioned as reasons for water logging hazard. Climatic phenomena were considered an important factor of coastal water logging. Among the studies, Tareq et al. (2018) conducted his study using geo-informatics and qualitative methods where he suggested to re-excavate the silted riverbed to solve the situation. The methodological framework and conceptualization of this study were similar but the locational characteristics and geomorphological attributes were quite different as Dhaka is a highly urbanized city and the capital of Bangladesh.

Since Dhaka has been undergoing a rapid unplanned urban development in the last two decades. As a result, the city has exceeded its carrying capacity due to extensive urban migration and along with untenable development resulting in several hazards. The development plan has not been implemented in a holistic and integrated way. Moreover, there is no integration within the different institutions of the interconnected neighborhood domains while deploying the interventions. WASA and the two City Corporations have mostly implemented their development projects individually without coordination. Also, the economic gap between rich and poor has been noticeably increasing, with the floating and informal population being deprived of the basic amenities; hence, the higher population density neighborhood areas always suffer from water logging hazards. Furthermore, 70 to 75% informal settlements are observed in the high to very high water logging vulnerable zone. This scenario demarcates that informal settlements are less prioritized while initiating a development plan. Also, almost 70% of poorly structured houses are found in high to very high water logging susceptible zone. These household members are the worst sufferers of the water logging hazard due to problems arising from floor dampness. The growing expansion of built-up areas has not been properly planned resulting in submersion of these areas during heavy rainfall.

Thiele-Eich et al. (2015) analyzed a trend of water level and flooding in Dhaka for the past 100 years and their findings suggested that minimum surface water levels have decreased by 0.71 to 0.61 m. While the magnitude and duration of the flood have reduced, the frequency of extreme flood events has increased in Bangladesh. Nevertheless, the study could not conclusively show a direct link with rise in mortality or higher morbidity rate due to extreme flood, but the relative risk of death was found to reduce with the decrease in water levels (Thiele-Eich et al., 2015). Inadequate draining capacity and inappropriate lining of pipes were considered the main cause of long-lasting floods in Dhaka (Pirumanekul & Mark, 2001). The geo-referenced model and simulation found that water levels in the street mainly cause urban flooding. The flood simulation model also implied that Shantinagar crossing had the highest inundation (55 cm/6 h) in 1997 and then Kakrail and Topkhana areas had the flood depth of 19 cm/6 h and 25 cm/12 h respectively. Dewan et al. (2004) delineated the flood extent map in Dhaka city using DEM, where 1988 and 1998 floods were also taken into consideration. During the 1988 flood, almost all the areas were inundated. However, owing to the construction of several embankments, areas in Dhaka city remain flood-free from river water but the rainfall-induced flooding has been very severe.

In several areas of Dhaka, the fast rate of urbanization has prompted water logging and urban floods. Moniruzzaman et al. (2020) in their study observed that from 1978 to 2018, there were 13.1%, 4.8%, and 7.8% decreases in agricultural land, green spaces, and aquatic bodies, respectively, and an estimated 22.1% rise in the built-up area in this region. Besides, they also found that the area with very high runoff has expanded from 74.24 km^2^ (24.44%) in 1978 to 174.23 km^2^ (57.36%) in 2018. It indicates that the land use land cover change, especially the decreasing trend of green spaces and water bodies of the study area, refers the significant rise in water logging frequency and intensity. Besides, regarding the rainfall pattern, September 2004 had seen the highest daily precipitation of 341 mm/day. And the annual maximum daily rainfall variation (1990–2018) ranges between 50 and 180 mm, with the exception of 2004 and 2009, when it exceeds 300 mm (Moniruzzaman et al., 2020).

Dewan et al. (2007) illustrated a hazard map that shows that a major portion of Dhaka city was located within a moderate to very high hazard vulnerable zone, especially the suburb areas which have become urbanized in 2010. Masood and Takeuchi (2012) found in their study that about 60% of eastern Dhaka regularly submerged in water every year in monsoon due to absence of flood embankment. Due to a lack of proper drainage system, water became clogged down for several hours. The study findings also support this outcome. Besides, Rashid et al. (2007) conducted a research on slum dwellers of Mirpur and Vasantek neighborhood areas. The study found that the respondents from Vasantek experienced flooding inside their homes more often and the inundation depth was higher in Vasantek areas than Mirpur areas. It also revealed that all slum dwellers were being exposed to mosquito-borne diseases due to the long-lasting floodwater.

In the urban context, Datta and Mandal (2017) stated that traffic congestion and water logging are the worst problems in Dhaka city. The study found a significant loss of natural water bodies across the city. They suggested forming a “blue network” within the city to solve the transportation and water logging problem. Besides, Subrina and Chowdhury (2018) conducted a study in Dhaka city to identify the causations and evaluate its impact based on internet open-source data and information. Population growth, unplanned development, the disappearance of natural drainage systems and green spaces, topography, waste management, and drainage capacity were identified as the major reasons for water logging. The findings of this study which were derived by utilizing open-source data were quite analogous to Subrina and Chowdhury’s study in the Dhaka context. The study recommended macro-scale solutions, including retrieval of canal networking and developing urban fringe areas. Lastly, the poor drainage network, dumping of waste everywhere resulting in the hindrance of water flow as well as clogging down the existing drainage channels. Additionally, absence of sufficient canals and reservoirs to hold extra water, pollution of surrounding rivers, etc. were identified as the main reasons behind worsening the water logging situation.

While the present study was able to identify and evaluate the water logging hazard in a highly urbanized area and analyze the risk by overlapping the infrastructure and demographic attributes through the lens of the GIS-RS approach in a temporal extent, a huge strength, it had some limitations too. The main limitation of the study was the unavailability of high-resolution satellite images. Also, we could not utilize the high-resolution DEM data. It could have more accurate if we used the high-resolution intra-annual satellite imageries. Updated socio-demographical dataset was also not available for use. Inadequately published literature and lack of data were considered the major drawback, which would have further enriched the study. Furthermore, it was not possible to acquire the groundwater and flooding data for the study because of its unavailability in a large capacity.

## Conclusions

The present research demarcated the water logging hazard zones in Dhaka city through an integrated GIS-remote sensing method and has shown the spatial distribution of the water logging vulnerabilities over the indicators of slum, population density, housing types, and floor materials. The results of the study suggest that the south and south-western part of the Dhaka city are comparatively susceptible to water logging hazards as these areas lie on highly to very highly vulnerable zones. Moreover, the slums were densified in the “very high” waterlogged areas. The waterlogged hazard and the vulnerability maps may possibly be used by planners, policymakers, and local people for urban development, water resources management, drainage network development, infrastructure expansion and housing management, etc. An inclusive and integrated approach needs to be included in the future development plans of all sectors to make it sustainable. It is also essential to give extra attention to the lower elevated areas. Informal settlement areas should also be specially focused while initiating an intervention. A proper drainage system needs to be established and the existing canals should also be well maintained and re-excavated regularly to enhance the retention capacity. The identified water logging hazard zones will also be useful for designing future projects in the urban neighborhood context and planning for an healthy city. The applied integrated GIS and remote sensing method can also be efficiently used for sustainable water resource management purposes.

## Data Availability

The datasets generated and analyzed during the current study are available from the corresponding author on reasonable request.

## Footnotes

1 According to Census of Slum Areas and Floating Population 2014, slum is a cluster of compact settlements of five or more households which generally grow very unsystematically and haphazardly in an unhealthy condition and atmosphere on government, private vacant land, or owner-based household premises.

2 NDVI = [Band 5 − Band 4]/[Band 5 + Band 4].

3 NDWI = [Band 2 − Band 1]/Band 2 + Band 1].

4 NDMI = (Band 4 − Band 5)/(Band 4 + Band 5).

5 The Raster Calculator tool allows to create and execute map algebra expressions and calculations like other geoprocessing tools. It can be used in Model Builder to maintain workflows in an integrated way.

6 Pacca is a housing type which is designed to be solid and permanent, typically made of brick with or without cemented. Semi pacca house is in between Pacca and Kacca such as the wall has made up of pacca materials but the roof is made up of tin, etc.

7 Kacca is narrow and built of mud brick or other temporary materials which is cheap and easy to make. Jhupri is a temporary house or shades mainly found in roadside, train or bus stations which is made of polythene, straw, and cheap plastic materials.
